# The Relationship between Perceived Stress and Marital Satisfaction in Couples with Infertility: Actor-Partner Interdependence Model

**DOI:** 10.22074/ijfs.2019.5437

**Published:** 2019-01-06

**Authors:** Saman Maroufizadeh, Mostafa Hosseini, Abbas Rahimi Foroushani, Reza Omani-Samani, Payam Amini

**Affiliations:** 1 Department of Epidemiology and Biostatistics, School of Public Health, Tehran University of Medical Sciences, Tehran, Iran; 2 Department of Epidemiology and Reproductive Health, Reproductive Epidemiology Research Center, Royan Institute for Reproductive Biomedicine, ACECR, Tehran, Iran

**Keywords:** Actor-Partner Interdependence Model, Infertility, Marital Satisfaction, Stress

## Abstract

**Background:**

Infertility, one of life’s great stressors, may adversely affect marital satisfaction. No studies have in- vestigated the relationship between perceived stress and marital satisfaction at the dyadic level. The current study assessed the actor and partner effects of perceived stress on marital satisfaction in husband-wife dyads using an in- novative dyadic analysis approach, the Actor-Partner Interdependence Model (APIM).

**Materials and Methods:**

In this cross-sectional study, we recruited a total of 141 infertile couples. Marital satisfac- tion and stress were assessed using the ENRICH Marital Satisfaction Scale (EMS Scale) and Perceived Stress Scale-4 Item (PSS-4), respectively. Dyadic data have been analysed by the APIM approach, with distinguishable dyads. In this approach, actor effect is the impact of a person᾽s perceived stress on his/her own marital satisfaction. Partner effect is the impact of a person's perceived stress on the partner᾽s marital satisfaction.

**Results:**

Both men and women’s perceived stress exhibited an actor effect on their marital satisfaction (β=-0.312, P<0.001, β=-0.405, P<0.001, respectively). Women’s perceived stress had a negative relationship to the marital satis- faction of their partner (β=-0.174, P=0.040). Although the partner effect of men’s perceived stress on woman’s marital satisfaction was not significant (β=-0.138, P=0.096), women whose husbands had higher levels of stress were more likely to have poorer marital satisfaction. Both actor and partner effects of perceived stress on marital satisfaction were similar among men and their wives.

**Conclusion:**

The findings of this study have highlighted that marital satisfaction in patients with infertility was in- fluenced by not only their own perceived stress, but also their spouses’ perceived stresses. Therefore, psychological interventions that target a reduction in perceived stress and enhancement of marital satisfaction in the context of infer- tility should treat the couple as a unit.

## Introduction

Infertility is medically defined as “the failure to achieve a clinical pregnancy after 12 months or more of regular unprotected sexual intercourse” (1). It is a public health concern that affects 9% of reproductive-aged couples worldwide (2). Infertility has been ranked as one of the great stressors in life and has a considerable impact on a person’s quality of life (3, 4). Infertility is negatively related to personal and marital health among infertile couples since it signifies one’s loss of ability to achieve parenthood (5). Infertile people experience more stress related to both infertility as a disease and its treatments when compared to fertile people (6). In addition, numerous researches have shown a negative association of stress with marital satisfaction (7, 8) and a relationship to a range of adverse health outcomes (9). For these reasons, this concept has received increased attention in marital studies in recent years. Studies have focused on different types of stress (e.g., internal vs. external, minor vs. major, and chronic vs. acute) and two key theoretical models (family and couples’ stress models). They have indicated that the role of stress is detrimental to the quality and longevity of a relationship (10).

Many of the phenomena studied by scientists in social and behavioural sciences are dyadic in nature and include research on man-woman dyads and parent-child dyads. The observations that arise from such designs are interdependent rather than independent; however, in this case, independence refers to independence from dyad to dyad (11,12). Statistically, conventional parametric statistics developed for independent individuals are not appropriate for non-independent observations. Instead, the non-independence due to the dyadic nature of data must be taken into account when relationships are examined. An example of non-independence is the characteristic or behaviour of one person that affects his or her partner’s outcomes; therefore, an analysis that takes non-independence into account is required. The Actor-Partner Interdependence Model (APIM), an innovative dyadic analysis approach, simultaneously estimates the effects an individual’s characteristics and the partner’s characteristics on an outcome variable. The APIM approach uses the dyad, not the individual, as the sampling unit. This approach provides separate, but simultaneous estimates of actor and partner effects (12). The actor effect measures the degree to which one’s own characteristics impacts his/her own outcomes, whereas the partner effect measures the degree to which one individual is influenced by the other individual or the partner.

Most studies that investigate the relationships between psychological distress and marital satisfaction in couples with infertility use the individual as the unit of analysis. Although valuable, these researches fail to show the impact that partner distress has on individual marital satisfaction. Since infertility is a shared problem, it is particularly relevant to examine the impact of partner distress (13). Perceived stress by the husband or wife does not only affect his/her own marital satisfaction, but also their partner's marital satisfaction. Therefore, the current study has aimed to examine whether differences existed in the levels of perceived stress and marital satisfaction between men and women dyads with infertility. We also used the APIM approach to elucidate and differentiate actor effects and partner effects of perceived stress on marital satisfaction.

## Materials and Methods

### Participants and study design

This was a cross-sectional study of a sample of couples with infertility from Tehran, Iran. Patients were recruited from the Infertility Treatment Centre of Royan Institute, a referral centre for infertility treatment in Tehran, Iran (14). The data were collected using the convenience sampling method between February and May 2017. Couples who met the following criteria were included in the present study: i. Married and in a heterosexual relationship, ii. Willingness to participate in the study, iii. Presence of fertility problems, iv. Age >18 years, and v. Ability to read, write, and comprehend Persian. The couples with infertility were asked to fill out the questionnaires separately from each other and refrain from discussing their answers. In total, 141 couples with infertility agreed to participate and completely filled out the questionnaires (response rate: 82.3%).

### Ethical consideration

The Ethics Committee of Tehran University of Medical Sciences, Tehran, Iran, approved this study. The participants were informed of the aim of the study and were assured of confidentiality. After signing a consent form and agreement to participate, the couples with infertility completed the questionnaires.

### Questionnaires 

#### Ten item ENRICH Marital Satisfaction Scale (EMS Scale)

The ENRICH Marital Satisfaction Scale (EMS Scale) is a 10-item self-report instrument designed to measure marital satisfaction (15). Each item is scored on a 5-point Likert scale as follows: 1 (strongly disagree), 2 (moderately disagree), 3 (neither agree nor disagree), 4 (moderately agree), and 5 (strongly agree). Total scores range from 10 to 50; higher scores are indicative of greater marital satisfaction. The Persian version of the EMS Scale has been shown to have good psychometric properties (16). For this study, the Cronbach’s alpha coefficient of the EMS Scale was 0.771.

#### Perceived Stress Scale-4 item (PSS-4)

The Perceived Stress Scale-4 item (PSS-4) is a short form of the PSS that measures the degree to which situations in one’s life over the last month are appraised as unpredictable, uncontrollable, and overloaded. Each item is scored on a 5-point Likert scale that ranges from 0 (never) to 4 (very often). Total scores range from 0 to 16, with higher scores indicating higher levels of stress (17). The Persian version of PSS has been shown to have good psychometric properties (18, 19). For this study, the Cronbach’s alpha coefficient of the PSS-4 was 0.572.

### Statistical analysis

Comparison of demographics characteristics, perceived stress, and marital satisfaction for husbands and wives were made using the McNemar test and paired sample t test. Pearson’s correlation coefficient was used to examine the correlation among the study variables.

**Fig.1:**
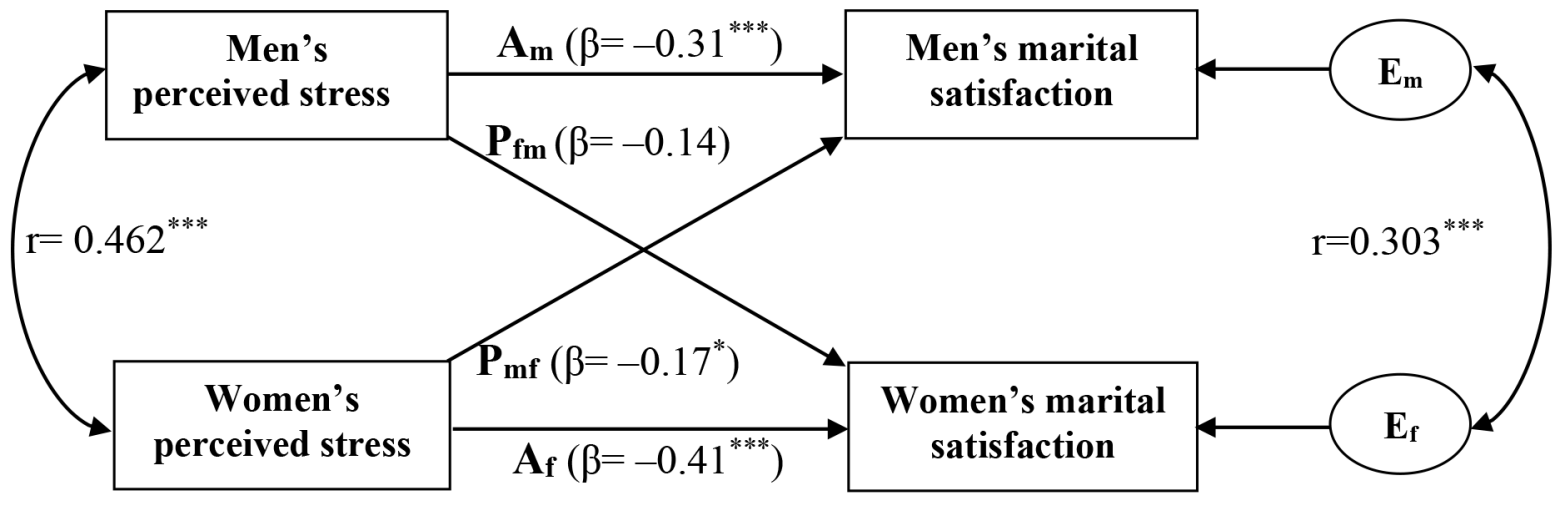
Actor-Partner Interdependence Model (APIM) of perceived stress and marital satisfaction. E_m_; Actor effect of husband’s perceived stress on his own marital satisfaction, E_m_; Actor effect of wife’s perceived stress on her own marital satisfaction, P_fm_; Partner effect of the husband’s perceived stress on his wife’s marital satisfaction, P_mf_; Partner effect of the wife’s perceived stress on the husband’s marital satisfaction, E_m_ and E_f_; Residual errors on marital satisfaction for men and women, respectively, ^*^; P<0.05, and ^***^; P<0.001.

We used the APIM with distinguishable dyads to determine the impact of husbands’ and wives’ perceived stresses on their own marital satisfaction, as well as their spouse’s marital satisfaction (12). Figure 1 depicts the APIM of a husband-wife dyad in which there are two variables from each in the dyad: perceived stress (independent variable) and marital satisfaction (outcome variable). The husband’s level of marital satisfaction is affected by his own level of perceived stress (actor effect, E_m_) and by his wife’s perceived stress (partner effect, P_mf_). Similarly, the wife’s level of marital satisfaction is influenced by her own perceived stress (actor effect, E_m_) and her husband’s perceived stress (partner effect, P_fm_). There are two important correlations in the model. The curved line that connects the independent variables indicates how similar the partners are on the predictor variables and the correlation between the error or residual terms (E_m_ and E_f_), which represents the non-independence that is not explained by the APIM.

Three different methods can be used to estimate the APIM: pooled regression modelling, multilevel modelling, and structural equation modelling (SEM). According to Kenny et al. (12), SEM with distinguishable dyads is the simplest data analytic method to estimate the APIM. The SEM approach involves estimating the APIM parameters as they appear in the model presented in Figure 1. Based on the dyad-level structure, there are two linear equations:

$$Y_{m}=A_{m}X_{m}+P_{\mathrm{mf}}X_{f}+E_{\mathrm{m’}}$$

$$Y_{f}=A_{f}X_{f}+P_{\mathrm{fm}}X_{m}+E_{\mathrm{f ’}}$$

where Y_m_ is the husband’s marital satisfaction, Y_f_ is the wife’s marital satisfaction, X_m_ is the husband’s perceived stress, and Xf is the wife’s perceived stress. In the first equation, E_m_ refers to the effect of the husband’s perceived stress on his own level of marital satisfaction (actor effect) and the partner effect, P_mf_, is the effect of the wife’s perceived stress on her partner’s marital satisfaction. Since the dyad is the unit of analysis, the sample size in this analysis is the number of couples (n=141). 

A useful attribute of SEM approach is that it allows model constraints to be placed and tested in the APIM framework. For example, this approach can test whether the husband’s actor effect is equal to the wife’s actor effect (E_m_=E_m_) and subsequently measure the degree to which this constraint significantly worsens the model fit (12, 20). The equality constraint test has been used to compare actor effects for men and women by examination of the chi-square difference test. If the chi- square difference test is statistically significant, it indicates that the actor effects for men and women cannot be the same. 

In order to compute a chi-square difference test, the difference of the chi-square values of the two models (constrained and unconstrained) in question is taken as well as the difference of the degrees of freedom.

$$\chi_{\mathrm{diff}}^{2}=\chi_{\mathrm{constrained}}^{2}-\chi_{\mathrm{unconstrained}}^{2}$$

$${\mathrm{df}}_{\mathrm{diff}}={\mathrm{df}}_{\mathrm{constrained}}-{\mathrm{df}}_{\mathrm{unconstrained}}$$

In the current study, all preliminary analyses were performed using IBM SPSS Statistics for Windows, version 22.0 (IBM Corp., Armonk, NY, USA). APIM analysis was performed using Mplus software version 6.12 (Muthén and Muthén, Los Angeles, CA, USA).

## Results

### Characteristics of dyads for men and women

The demographic and clinical characteristics of the men and women dyads are presented in Table 1. On average, husbands were 5.10 years older than their wives (t(140)=12.88, P<0.001) and they had a similar education level as their wives ((χ^2^_(1)_=2.56, P=0.109). The mean duration for marriage was 7.37 ± 4.40 years and for infertility, it was 4.85 ± 3.76 years. The causes of infertility were as follows: male factor (36.2%), female factor (21.3%), both (19.1%), and unexplained (23.4%). The majority of the couples had primary infertility (72.3%) and no history of abortion (76.6%). Half experienced at least one failure in previous ART treatments.

### Marital satisfaction and perceived stress in dyads for men and women

As presented in Table 2, the marital satisfaction scores for the husbands and their wives were similar (t_(140)_=0.09, P=0.925), but the women had greater perceived stress compared to their husbands (t_(140)_=2.06, P=0.042).

Perceived stress in husbands was correlated with both their own marital satisfaction (r=-0.393, P<0.001) and their wives’ marital satisfaction (r=-0.325, P<0.001). Perceived stress in wives was also correlated with both their own marital satisfaction (r=-0.469, P<0.001) and the husband’s marital satisfaction (r=-0.319, P<0.001) (Table 3).

**Table 1:** Demographic and clinical characteristics of the men and women dyads (n=141 couples)


Variable		Men	Women	Test statistic	P value

Age (Y)		34.92 ± 6.35	29.82 ± 6.00	t_(140)_=12.88	<0.001
Educational level				χ^2^_(1)_=2.56	0.109
	Non-academic	96 (68.1)	85 (60.3)		
	Academic	45 (31.9)	56 (39.7)		
Duration of marriage (Y)		7.37 ± 4.40	-		
Duration of infertility (Y)		4.85 ± 3.76	-		
Cause of infertility					
	Male factor	51 (36.2)	-		
	Female factor	30 (21.3)	-		
	Both	27 (19.1)	-		
	Unexplained	33 (23.4)	-		
Failure of previous treatment					
	No	71 (50.4)	-		
	Yes	70 (49.6)	-		
History of abortion					
	No	108 (76.6)	-		
	Yes	33 (23.4)	-		
Type of infertility					
	Primary	102 (72.3)	-		
	Secondary	39 (27.7)	-		


Data are presented as mean ± SD and n(%).

**Table 2:** Comparisons in marital satisfaction between men and women, and perceived stress (n=141 couples)


Variable	Men	Women	t_(140)_^a^	P value

Perceived stress	5.83 ± 2.80	6.33 ± 2.81	2.06	0.042
Marital satisfaction	39.31 ± 6.56	39.26 ± 6.70	0.09	0.925


^a^; Test statistic. Values are presented as mean ± SD.

**Table 3:** Correlations among predictors and outcomes in dyads for men and women (n=141 couples)


Variable	1	2	3	4

1 Perceived stress in males	1			
2 Marital satisfaction in males	-0.393	1		
3 Perceived stress in females	0.462	-0.319	1	
4 Marital satisfaction in females	-0.325	0.423	-0.469	1


All correlations were significant at the 0.001 level.

### Impact of perceived stress on marital satisfaction at the dyadic level

According to Table 4 the results for the APIM indicated that the husband’s perceived stress as well as the wife’s perceived stress exhibited an actor effect on their marital satisfaction (ß=-0.312, P<0.001, ß=-0.405, P<0.001, respectively). With regard to partner effects, only the woman’s perceived stress had a partner effect on the husband's marital satisfaction (ß=-0.174, P=0.040). Although the partner effect of the husband’s perceived stress on the wife's marital satisfaction was not significant (ß=-0.138, P=0.096), women whose husbands had higher levels of stress were more likely to have poorer marital satisfaction.

We used the equality constraint tests to compare actor effects as well as partner effects for men and women by examination of the chi-square difference test. Constraining the actor effects to be equal did not significantly worsen the model fit ((χ^2^_(1)_=0.60, P=0.437), which indicated that the actor effects of perceived stress on marital satisfaction were similar for men and women. The same findings were also observed in the partner effects (.2 (1)=0.07, P=0.795).

**Table 4:** Actor and partner effects of perceived stress on marital satisfaction in couples with infertility (n=141)


	Men			Women		
	β (95% CI)	t^a^	P value	β (95% CI)	t^a^	P value

Actor’s stress	-0.31 (-0.48, -0.15)	3.77	<0.001	-0.41 (-0.56, -0.25)	5.22	<0.001
Partner’s stress	-0.17 (-0.34, -0.01)	2.05	0.040	-0.14 (-0.30, 0.02)	1.67	0.096


^a^; Test statistic.

## Discussion

To the best of our knowledge, this study is the first of its kind to use the APIM to examine the impact of actor and partner stress on marital satisfaction in a sample of couples with infertility. Although the majority of researches that have examined psychological distress and marital satisfaction both in infertile and fertile couples assessed the actor effect of stress on marital satisfaction, there are increasing calls to investigate the partner effect of these variables. Since infertility is a shared problem within the couple, both men and women need to be involved and considered as a dyad.

As expected, perceived stress among the wives was higher than their husbands, which suggested that women tend to perceive stressful life events as less controllable than men and generally seem to be more affected in terms of negative life consequences. Another explanation for this difference could be that generally, particularly in Iran or Middle Eastern countries, childbirth is considered the women's duty and infertility is considered a disease in women. The burden of infertility is mostly on women. This result has supported the findings of previous studies (21, 22). Consistent with a study by Peterson et al. (22), marital satisfaction was unrelated to gender. In a study conducted among couples with infertility in Poland, women had worse marital satisfaction than men (23).

The current study has found the actor effect of perceived stress on marital satisfaction. In other words, the greater level of stress that is perceived by either men or women contributes to lower marital satisfaction for themselves. This is in line with a study of patients with infertility in France, in which the predictive effects of infertility-related stress on both emotional and marital distress have been confirmed (21). Additionally, in a review based on 24 empirical studies, different types of stressors were associated with marital satisfaction and its longevity (10).

The most important finding of the current study was the link between an individual’s perceived stress and their partner’s marital satisfaction. In accordance with our expectation, we found that a woman’s perceived stress negatively impacted the man’s marital satisfaction. Contrary to our expectation, our study did not confirm a strong partner effect of a man’s perceived stress on marital satisfaction, although the impact of the husband’s perceived stress on his wife’s marital satisfaction was marginal.

Our results indicated that the actor effects and partner effects of perceived stress on the marital satisfaction were similar for both men and women. Although the levels of perceived stress differed between men and women, the associations between stress and marital satisfaction were not substantially different between them. This finding might indicate that both members of couples with infertility share a similar mechanism through which perceived stress influences marital satisfaction.

This study has several limitations that should be considered when interpreting the results. First, the generalizability of the results might be affected by the fact that it was a single-centre study with a relatively small sample size. Second, because of the cross-sectional nature of the study design, causal inferences could not be made. In addition, this study relied on self-reported data that might be prone to social desirability bias. Despite these limitations, this study has provided valuable information regarding the actors and partner effects of perceived stress on marital satisfaction in men-women dyads that experience infertility. 

## Conclusion

The findings demonstrate that partner effects are present in couples with infertility and support the idea that a person’s perceived stress can impact his or her partner’s marital satisfaction. Psychological interventions that target a reduction of perceived stress and enhancement of marital satisfaction in the context of infertility should treat the couple as a unit.
